# Effects of Early Lactation Milking Frequency in an Automated Milking System on Cow Performance

**DOI:** 10.3390/ani14162293

**Published:** 2024-08-06

**Authors:** Erin M. Kammann, Elizabeth A. French, Natalie S. Jozik, Wenli Li, Ryan S. Pralle

**Affiliations:** 1 Department of Animal and Dairy Sciences, University of Wisconsin-Madison, Madison, WI 53706, USA; kammann@wisc.edu; 2 US Dairy Forage Research Center, USDA-ARS, Madison, WI 53706, USA; elizabeth.french@usda.gov (E.A.F.); wenli.li@usda.gov (W.L.); 3 School of Agriculture, University of Wisconsin-Platteville, Platteville, WI 53818, USA; jozikn@uwplatt.edu

**Keywords:** milking frequency, transition period, automated milking systems, robotic milking, health, metabolism

## Abstract

**Simple Summary:**

The increasing use of automated milking systems (**AMS**) in the dairy industry has led to the necessity for changes in management practices implemented by producers. This study investigated the productive and metabolic impact of three versus six milkings per day (**MPD**) during early lactation on multiparous Holstein cows managed in an AMS. Mature cows (third and greater parity) with six MPD had greater milk fat production during the experimental phase (4 to 29 days in milk; **DIM**) and milk production during the carryover phase (30 to 90 DIM) than mature three MPD cows. The composition of milk fatty acids differed between MPD groups with cows, with three MPD having greater short-, medium-, odd- and branched-chain fatty acids during the experimental phase compared to cows with six MPD. Based on greater blood fatty acid and β-hydroxybutyrate concentrations, the mature six MPD cows may have been in a greater postpartum nutrient deficit and at greater risk of metabolic illness. Our findings suggest a need to strategically manage the MPD or nutrition of mature, early lactation dairy cows in an AMS.

**Abstract:**

Automated milking systems (**AMS**) are increasingly adopted for dairy cow production, promoting individualized cow management dependent on factors like lactation stage, age, and productivity. The study objective was to investigate the effects of early lactation milking frequency on cows milked via AMS. Multiparous Holstein cows blocked by parity and due date were randomly assigned to treatments (n = 8 per treatment): three (**3X**) or six (**6X**) milkings per day (**MPD**). The experimental phase (**EXP**) was defined as 4 to 29 days in milk (**DIM**). The AMS settings were programed so 3X cows were limited to three MPD while 6X cows were allowed six MPD. Afterwards was the carry over phase (**CO**) ranging from 30 to 90 DIM; all cows were allowed up to six MPD. Measurements by the AMS included bodyweight, milk yield (**MY**), and pellet intake. Weekly composite milk samples were analyzed for macronutrient composition and fatty acid (**FA**) profile. Coccygeal blood was sampled at 3, 8 ± 1, and 13 ± 1 DIM; concentrations of blood plasma analytes were quantified. Greater MPD was achieved for 6X cows versus 3X cows during EXP, but similar during the CO. Daily MY was non-separable during the EXP while 6X cows in their third or greater lactation group (**3 + LG**) had greater MY than 3X cows of the same LG during the CO. Milk fat content and 4% fat-corrected MY were both greater for 6X, 3 + LG cows during the EXP compared to 3X, 3 + LG cows. Milk FA methyl esters (**FAME**) proportions were different between MPD groups, with 6X, 3 + LG cows having the lowest short, even-chain FA from de novo or post-absorptive origin. Differences in analytes indicated that 6X, 3 + LG cows experienced metabolic stress and incorporated greater FA from adipose tissue. Greater early lactation MPD in AMS may shift cow nutrient partitioning to support greater production in 3+ parity cows.

## 1. Introduction

Milk production is an important economic output for dairy farms, making the ability to maximize milk yield per cow or to optimize milk output efficiency a priority for producers. Adjusting milking frequency is a relatively simple opportunity to improve milk yield. Dairy farms that milk cows using a conventional milking parlor system (**CMP**) where a whole group of cows are brought to be milked together, such as a herringbone, parallel, or carousel, typically employ 2 or 3 milkings per day (**MPD**) [1]. The average MPD for farms that use automatic milking systems (**AMS**) is between 2.5 and 3.0 However, MPD is not uniform across all lactating cows and can vary substantially based on lactation stage, productivity, and management strategies [2]. Research has generally reported greater milk production per cow with greater MPD [3,4,5,6,7]. Compared to two MPD cows, three MPD cows may produce 20% more milk [3,8]. At three or more MPD during early lactation followed by lower MPD from mid lactation onwards, the improvement to milk production may persist through the same lactation [4,5]. Wall and McFadden [9] estimated the economic benefit for cows that had four MPD for the first three weeks of lactation and two MPD for the rest of lactation to be USD 9294 net income per 100 cows per year based on a milk price of USD 12/cwt.

The improvement in milk production due to greater MPD is mediated by several mechanisms. As milk accumulates in the lumen of the alveoli, milk ducts, and udder cisterns until milking, it will lead to reduced milk secretion due to filling [8,10,11]. It is the physical removal of milk that stimulates milk secretion and with more MPD, the mammary gland can continue milk production by limiting the time the mammary gland is full between milkings [8,10,11]. The number of days at a higher number of MPD impacts different mechanisms that control milk yield, including number and metabolic activity of mammary gland secretory cells [8]. Short-term increases in MPD over one to seven days affect regulatory activities including feedback inhibition of lactation, tight junctions, and apoptosis [8,12]. During long-term increases in MPD, between a week to a couple weeks, the mammary gland adapts to greater cellular activity with cellular differentiation [8,12]. An increase in mammary parenchyma by stimulating cell proliferation is seen when MPD is increased for multiple weeks to months [8,12]. These mechanisms contribute to the greater milk secretion observed with increasing MPD [12].

A variety of investigations into different MPD durations within lactation have tested for milk yield improvement, spanning from the whole lactation to as short as the first 7 days in milk (**DIM**) [4,6,9,13,14]. Eslamizad et al. [6] compared a complete lactation of 3 MPD, 6 MPD, and a crossover from 6 MPD for the first 90 DIM to 3 MPD (**6–3 MPD**) for the remainder of lactation and reported that milk production was greater for 6 MPD and 6–3 MPD cows compared to three MPD cows. The 6–3 MPD cows produced more milk, fat-corrected milk (**FCM**), and energy-corrected milk (**ECM**) than the six-MPD group during the first two months of lactation. However, the 6–3 MPD cows were not as persistent, and a no carryover improvement to production was observed [6]. The authors suggested that the 6–3 MPD cows experienced a lactation curve collapse due to being less mature than the 6 MPD cows, resulting in less energy per unit of BCS loss being released and a large negative energy balance (**NEB**) [6]. Other studies where MPD was greater during mid or late lactation had milk production improvement during the intervention; however, the greater production was not maintained after MPD was reduced [15,16,17]. Hale et al. [4], Dahl et al. [5], and Shoshani et al. [14] implemented greater MPD for the first three weeks of lactation. Compared to lower-MPD controls, the greater-MPD cows had greater milk yield during the first three weeks postpartum as well as a carryover effect of greater milk production for a portion of the remaining lactation. These data indicate that greater MPD during early lactation may have the greatest long-term impact of improved production.

Cows enter a NEB during periods when whole-body energy requirements exceed energy intake, such as early lactation [18,19,20]. This NEB is due to the increase in energy requirements of milk production and a relative lag in energy intake [18,19]. In response, cows will mobilize adipose tissue triglycerides, an endogenous energy source, as fatty acids (**FA**) to support lactation energy requirements. However, maladaptation to this response, such as saturating liver lipid complete oxidation and secretion pathways [18,21], can result in metabolic stress that may reduce milk production and other performance metrics [22,23,24,25]. For example, cows may develop the metabolic disorder hyperketonemia (**HYK**), an abnormally greater production of ketone bodies like β-hydroxybutyrate (**BHB**), which has a USD 289 total deterministic cost per case [19,26]. Indeed, cows with greater milk production are associated with greater tissue mobilization and risk of HYK [27,28].

Most controlled MPD research has utilized CMP. Few controlled studies have investigated the effects of increased MPD with AMS. With the increasing number of farms transitioning to or starting with AMS in place of CMP, there is a growing demand for controlled research using AMS due to differences in milking frequency and feeding practices [29]. For dairies that use CMP, cows have a set MPD with limited variation from day to day. Within AMS, cows have continuous access for milking, which can promote greater MPD and associated variation within and across cows. It is a common management practice to include a palatable pellet or concentrate in the AMS during milking to promote voluntary visits. Additionally, this pellet serves as a nutritive source and is often variably fed based on cow productivity [30,31,32]. Thus, observational investigations into the impact of MPD on productivity in AMS are often confounded by greater concentrate intake. This variation in interval, frequency, milk yield, and both concentrate and PMR intake that occurs when manipulating MPD withing AMS may yield different results than when manipulated in CMP.

We hypothesized that AMS cows with greater MPD during early lactation will have greater milk production and greater risk for metabolic stress. To test this hypothesis, we imposed two MPD treatments, three and six MPD, for the first month of lactation on cows milked by AMS. These MPD have been previously used in previous studies [6,14] to represent an average and above average MPD but have not been applied to cows in an AMS setting. The study objectives were to evaluate the effect of MPD on milk production and composition, blood biomarkers associated with metabolic illness, and rumen fermentation for cows milked by AMS.

## 2. Materials and Methods

### 2.1. Animal Housing and Management

Animal use and handling protocols for this research were approved by the University of Wisconsin—Platteville Animal Care and Use Committee (protocol #0131-2022). All cows on experiment were group housed in a freestall pen at the Pioneer Farm Dairy Enterprise (University of Wisconsin—Platteville, Platteville, WI, USA) with cows not on experiment. Cows had free access to water, the feed bunk, and laying space (mattress stalls bedded with wood shavings) and had access to an AMS (Lely Astronaut A5, Lely Industries N.V., Maassluis, The Netherlands). The pen stocking density was constant at 50 cows (including animals on experiment and not), 47 stall beds, about 100 cm of linear water space, and 33 m of feed bunk space.

### 2.2. Experimental Design

The eligibility requirement for cow enrollment was that they had completed their previous lactation while milked by an AMS. Multiparous Holstein cows (n = 16) were blocked based on expected calving date (10 d intervals, 6 levels) and randomly assigned to treatment (n = 8 cows per treatment). Treatments (**TRT**) included cows with three MPD (**3X**) or six MPD (**6X**). Both treatments included cows in their second or third and greater lactation distributed across the treatments as follows: 3X had five cows in their second and three cows in their third or greater lactation, while 6X had six and two, respectively. For the first three DIM, all cows had three MPD. At four DIM a fetching schedule was employed to help ensure cows achieved the targeted MPD for their treatment. Cows on 3X had their maximum MPD limited to three by the AMS software Lely T4C version 3.12 (Lely, Maassluis, The Netherlands); they were fetched daily at 0345, 1200, and 1900 h. Meanwhile, 6X cows were fetched at 0345, 0700, 1200, 1545, 1900, and 2300 h daily. These times were chosen to align with Pioneer Farm’s typical fetching and chore schedule. For either treatment, cows were not milked at the fetch time when their previous milking was <3 h previous. Cows remained on their respective treatments fetch schedule from 4 DIM to 29 DIM; we refer to this period as the experimental phase (**EXP**). After the EXP, all cows were allowed to visit the AMS voluntarily with a maximum limit of six MPD; farm staff did fetch cows twice per day at 6 am and pm when their milking interval exceeded 10 h per farm policy. Cows were observed from 30 DIM to 90 DIM, which we refer to as the carryover phase (**CO**). All cows were offered the same partial mixed ration and AMS pellet (Table 1). During the EXP, both groups of cows had the same amount of pellet feed provisioned, which increased as DIM increased until 21 DIM. On an as-fed basis, the initial pellet feeding rate was 2.16 kg/d and increased by ~275 g/d to 3.63 kg/d. At 21 DIM and throughout the CO, they were then switched to follow a feed table based on milk production.

### 2.3. Sample Collection

Concentrated feedstuffs, including AMS pellet, were sampled by delivered load. Bi-weekly forage samples, except for baleage, were collected. A composite of baleage core samples (~25% of bales) was collected. Forage dry matter was determined by drying in a forced air convection oven at 55 °C for 48 h. Dried forage samples were ground to pass through a 1 mm sieve and composited by month. Proximate analysis of monthly forage composites and concentrates were analyzed by a commercial lab (Rock River Laboratory, Watertown, WI, USA); these updated values were used to reconstruct the partial mixed ration (PMR) and total diet delivered (Table 1). Diet energy density was calculated assuming a DMI of 60 kg/d using commercia software (CNCPS v.6.55; NDS Professional v.3.9.11, RUM&N, Reggio Emilia, Italy). The PMR was fed once daily at approximately 0700 h each morning.

Blood samples were collected by venipuncture of a coccygeal vessel on 3, 8 ± 1, and 13 ± 1 DIM starting at approximately 0630 h. Samples were collected into 10 mL evacuated tubes containing lithium heparin (158 USP) as an anticoagulant (BD Vacutainer, Becton, Dickinson and Company, Franklin Lakes, NJ, USA). The samples were placed in ice until centrifugation at 2000× *g* and 5 °C for 15 min; plasma supernatant was aliquoted in triplicate into 1.5 mL microcentrifuge tubes and stored at −80 °C.

Composite milks samples were regularly collected for traditional macronutrient composition analysis and milk fatty acid analysis using an automatic sampler (Lely Shuttle, Lely Industries N.V.) connected to the AMS. Milk samples for macronutrient composition were collected weekly from cows during the EXP. All milkings from a 24 h period were collected into vials containing a drop (approximately 0.042 mL) of 2-bromo-2nitropropane-1,3-diol (Advanced Instruments Inc., Norwood, MA, USA) and refrigerated at 4 °C until shipped. Milk composition samples were analyzed by a dairy herd improvement association laboratory for fat, protein, lactose, solids not fat, and milk urea nitrogen (AgSource, Menominee, WI, USA). A second set of milk samples were collected at consecutive milkings over a 24 h period between 3 and 8 DIM, and 23 to 28 DIM. Samples were frozen at −20 °C until individual fatty acid analysis.

Rumen fluid was collected 2 h after feeding via esophageal tubing of cows on 3, 13 ± 1, and 23 ± 1 DIM. The first 250 mL were discarded to remove any contamination from saliva. A minimum of 500 mL was collected for subsampling with rumen fluid to be used for volatile fatty acid, ammonia, and total amino acids. Rumen fluid subsamples were strained through 4 layers of cheesecloth, acidified with 50% H_2_SO_4_ to reach 0.01 vol/vol, and stored at −20 °C until analysis.

### 2.4. Sample Analysis

Blood plasma from 3, 8 ± 1, and 13 ± 1 DIM had analytes quantified by colorimetric assay using an automated high volume chemistry equipment (Carysta HVC, Zoetis, Parsippany, NJ, USA; DiaSys, Holzheim, Germany) to assess the energy status and health of the cows. Analytes quantified in duplicate included: albumin (**ALB**; #1 0220 9917 923, Zoetis), aspartate transferase (**AST**; #1 2601 99 17 920, Zoetis), BHB (#1 3711 99 17 921, Zoetis), cholesterol (#1 1300 99 17 923, Zoetis), glucose (#1 2511 99 17 920, Zoetis), and FA (#1 5781 99 17 92, Zoetis). The assay for alanine transaminase (**ALT**; #1 2701 99 17 920, Zoetis) was performed in triplicate. Reagents for the respective assays were calibrated using TruCal Lipid (#1 3570 99 17 045, Zoetis) for FA, BHB Standard (#1 3700 99 17 030, Zoetis) for BHB, and TruCal U (#5 9100 99 17 063, Zoetis) for all other analytes as needed as per manufacturer guidelines. Manufacturer controls—TruLab L1 and 2 (#5 9020 99 17 065 and #5 9030 99 17 065, respectively, Zoetis) for FA and TruLab N and P (#5 9000 99 17 062 and #5 9050 99 17 062, respectively, Zoetis) for all other analytes—were analyzed daily prior to sample analysis as well as after assay calibrations. An aliquot of an arbitrary plasma pool was analyzed prior to sample analysis as an inter-assay control. All assays had inter-assay coefficients of variation (**CV**) less than 10% and sample median intra-assays CV less than 4% (Table S1).

Milk macronutrient composition was determined for composite milk samples through an outside commercial laboratory. Briefly, analysis of milk fat and milk protein began by heating all milk samples to 40 °C and individually mixed before analysis. It was then analyzed by Fourier Transform Infrared using the Foss MilkoScan FT + (Foss Analytical, Hillerød, Denmark) following instrument manufacturer’s instructions and and standardized methods [33]. Milk samples were analyzed on equipment calibrated weekly with 12 standards per the laboratory’s standard operation procedures.

Milk FA were extracted from whole milk in hexane isopropanol, base transmethylated with sodium methoxide, and quantified by Shimadzu GC with a fused-silica capillary column (SP-2560, 100 m × 0.25 mm i.d. with 0.2-μm film thickness; Supelco Inc., Bellefonte, PA, USA) and a flame ionization detector with hydrogen as the carrier gas [34]. The initial oven temperature was 140 °C, held for 5 min, and was increased by 4 °C/min to 240 °C and held for 30 min. Inlet and detector temperatures were 250 °C with a 100:1 split ratio. Constant gas flows were 15 mL/min for hydrogen carrier, 12 mL/min for detector hydrogen, 400 mL/min for detector airflow, and 12 mL/min for detector nitrogen plus carrier. Fatty acid peaks were identified using FAME standards (GLC 461, GLC 780, and pure *trans*-10, *cis*-12 CLA and *cis*-9, *trans*-11 CLA, NuChek Prep Inc., Elysian, MN, USA; Bacterial Acid Methyl Ester Mix, 47080-U, Sigma-Aldrich, St. Louis, MO, USA; and GLC 110 mixture, Matreya LLC., State College, PA, USA). Recovery of individual FA was determined using an equal weight reference standard (GLC 461; NuChek Prep Inc.). Correction factors for individual FA and calculation of milk FA yield were carried out as described by Rico and Harvatine [35].

Ruminal samples were thawed at room temperature and centrifuged (15,300× *g* for 20 min at 4 °C), and flow-injection analyses (Lachat Quik-Chem 8000 FIA; Lachat Instruments, Milwaukee, WI, USA) were applied to supernatants to determine ammonia, using a phenol-hypochlorite method (Lachat Method 18-107-06-1-A; Lachat), and total AA as leucine equivalents [36]. Organic acids in ruminal liquid samples were determined by HPLC [37].

### 2.5. Milk Production, AMS Raw Data, and Biometrics

Raw data were taken from the AMS software to collect each cow’s daily summaries for information on milk production, pellet-related variables, rumination minutes, eating minutes, and body weight. For body weight, a three-day rolling average was used to reduce variability [38]. Body weight change was calculated for each phase by taking the difference between the body weight at the start and end of the same phase. Pellet related variables were named and defined as the following: pellets provisioned was the total amount of pellet feed a cow could receive in a day, pellets offered was the amount of pellet feed the AMS dispensed to the cow, and residual pellets was the difference between the pellet provisioned and offered amounts. The variable concentrate per 45 kg milk, which is the amount of pellet offered to the cow for every 45 kg or milk produced, was also collected from the AMS and was used to calculate concentrate per 45 kg EMC by dividing the concentrate per 45 kg milk by ECM.

### 2.6. Data Analysis

All data analysis was performed using the SAS software (version 9.4, SAS Institute Inc., Cary, NC, USA) procedures UNIVARIATE and GLIMMIX; data from the EXP and CO were analyzed separately. Normality was assessed for continuous response variables using the Shapiro-Wilk test; variables that were non-Gaussian (*p* ≤ 0.05) underwent systematic evaluation for data transformations to became empirically normal (*p* > 0.05). Then, data was analyzed using generalized linear mixed models developed using a systematic procedure as described previously [39]. Initial models included the fixed effects of TRT, lactation group (**LG**, 2 versus 3+ parity), DIM, and all interactions as well as the random effects of cow and block (expected calving date). The response variable was the variable of interest including production, pellet, and analyte variables. For milk samples, the weighted average for macronutrient composition across a 24 h period was used to determine daily component composition and yields. These daily amounts were then used for calculating daily values for energy-corrected milk (ECM; Equation (1)) [40], 4% fat-corrected milk (FCM; Equation (2)) [41], and milk energy (MilkE; Equation (3)) [42].

Equation (1):

ECM, kcal/kg milk = 0.327 × Milk (kg) + 12.95 × Milk Fat (kg) + 7.2 × Milk CP (kg)(1)

Equation (2):

4% FCM, kcal/kg milk = 0.4 × Milk (kg) + 15 × Milk Fat (kg)(2)

Equation (3):

(3) $$MilkE,Mcal/kgmilk=9.29\times \frac{Fat(kg)}{Milk(kg)}+5.85\times \frac{TP(kg)}{Milk(kg)}+3.95\times \frac{Lactose(kg)}{Milk(kg)}$$

The covariate of previous mature equivalent 305-day milk was used with all milk production related variables. Additionally, linear mixed models (**LMM**) included repeated measure of cow across DIM when necessary; variance covariance structure (variance component, first order autoregressive, or heterogeneous first order autoregressive) was selected based on the lowest model BIC. If the TRT × LG × DIM effect had low evidence (*p* > 0.10), the interaction was removed as long as the model BIC improved. For LMM, conditional studentized residuals were subjectively evaluated by plotting (i.e., linear predictor × studentized residuals, studentized residual quantile-quantile plot, effect × studentized residuals). When studentized residuals had a non-Gaussian distribution or unequal variance across linear predictors, modeling heterogeneous variance or alternative variance-covariance structures were investigated. Potential heterogeneous groups were determined based on plotting model variables by studentized residuals. Several heterogeneous groups were investigated for each LMM; the reported LMM had the lowest BIC or improved studentized residual plots. Non-linear mixed models were evaluated for overdispersion; all models had a dispersion < 1.50. The final mixed model for each response, including distribution and link function, is detailed in Table S2. Significant evidence for fixed effect differences was declared at *p* ≤ 0.05 and marginal evidence at 0.05 < *p* ≤ 0.10. Whenever TRT interacted with LG or DIM (*p* ≤ 0.10), simple (or slice) effect comparisons were made between TRT within the interacting factor (LG or DIM). Pairwise and simple effect comparisons were corrected by Bonferroni’s adjustment. Least squares means are expressed in text as the mean ± SEM except when a continuous variable was transformed to promote normality; in those cases, the back-transformed mean (95% confidence interval) was reported.

## 3. Results

**Figure 1:**
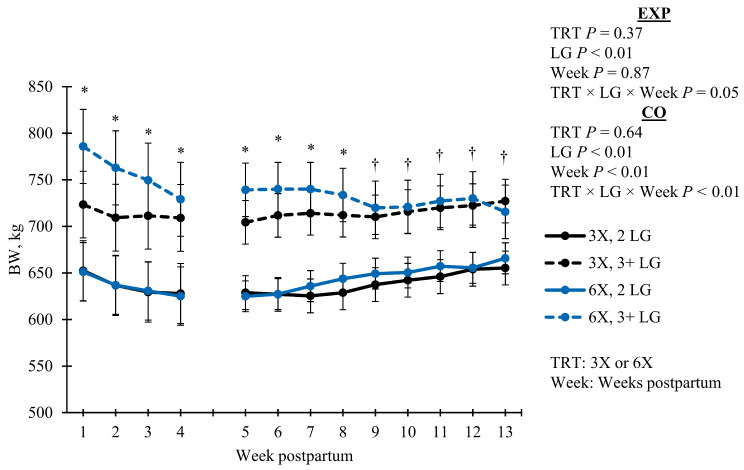
Body weight (BW) by wk postpartum across the experimental (EXP; 4 to 29 days in milk) and carryover (CO; 30 to 90 days in milk) phase for cows milked 3 (3X) or 6 times per day (6X). Average BW was calculated using a rolling three-day average. Lactation groups are second parity (2 LG) and third or greater parity (3 + LG). Error bars represent the standard error of the means. Simple effect comparisons of milking frequency treatment within the 3 + LG are indicated when there was significant (*; *p* ≤ 0.05) or marginal evidence (†; 0.05 < *p* ≤ 0.10) for a difference.

Milkings per day was greater during EXP (*p* < 0.01, Table 2) for 6X cows (5.0 ± 0.2 MPD) than 3X cows (3.1 ± 0.1 MPD) by design. However, MPD was similar (*p* = 0.55, Table 2) between TRT during CO (3.7 ± 0.2 and 3.6 ± 0.2 for 3X and 6X cows, respectively). Parity was similar (*p* = 0.85, Table 2) between TRT. Cow body weight (**BW**) differed based on the TRT, LG, and DIM interaction (*p* = 0.05) during EXP and CO (Figure 1). During the EXP, BW decreased as lactation progressed; however, 6X cows in the 3 + LG lost more BW than 3X cows of the same LG (simple effect *p* < 0.05). For the CO, most groups increased in BW over time, except for the 6X in the 3 + LG; those cows continued to decrease in BW while still maintaining greater BW than the 3X cows in 3 + LG (Figure 1). Body weight change was similar between TRT for EXP (*p* = 0.83) and CO (*p* = 0.11; Table 2).

**Figure 2:**
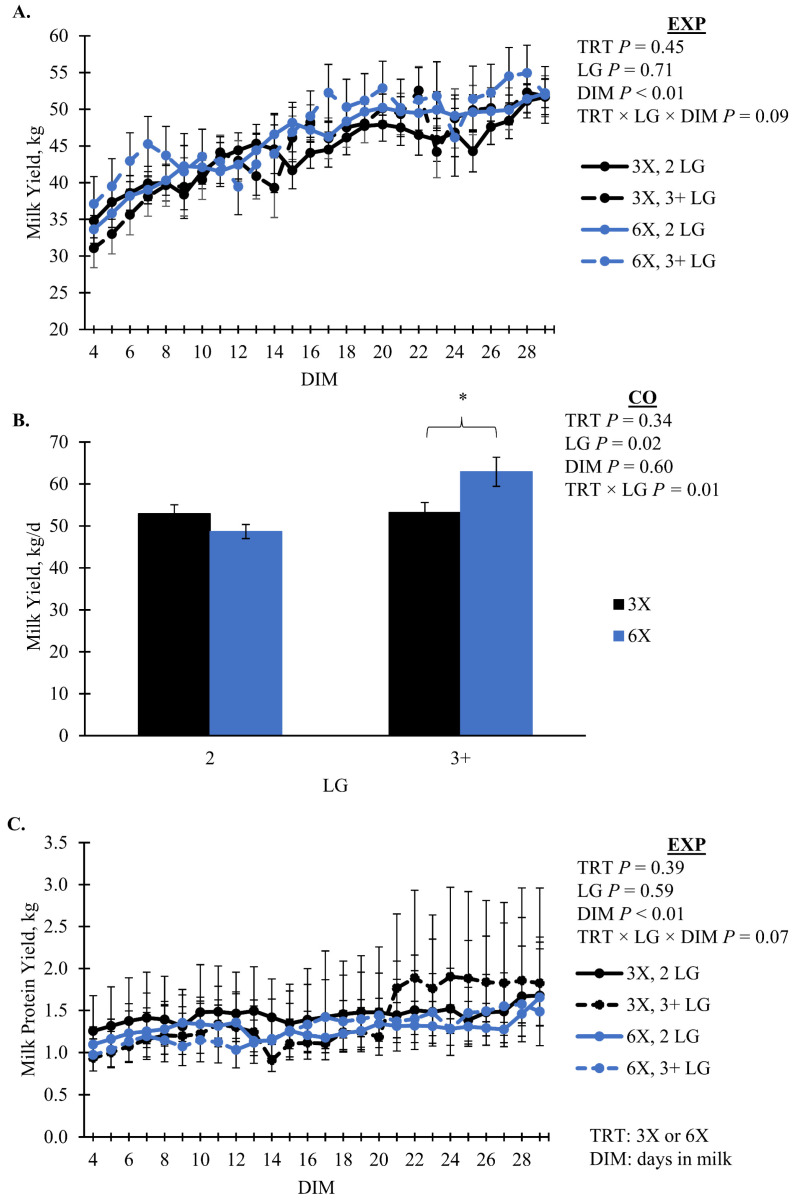
Daily milk yield during the (**A**) experimental (EXP; 4 to 29 days in milk, DIM) and (**B**) carryover (CO; 30 to 90 DIM) phases along with (**C**) daily milk protein yield during the EXP phase for cows milked 3 (3X) or 6 times per day (6X). Lactation groups are second parity (2 LG) and third or greater parity (3 + LG). Error bars represent the standard error of the means. Simple effect comparisons of milking frequency treatment within the 3 + LG are indicated when there was significant evidence (*; *p* ≤ 0.05) for a difference.

**Figure 3:**
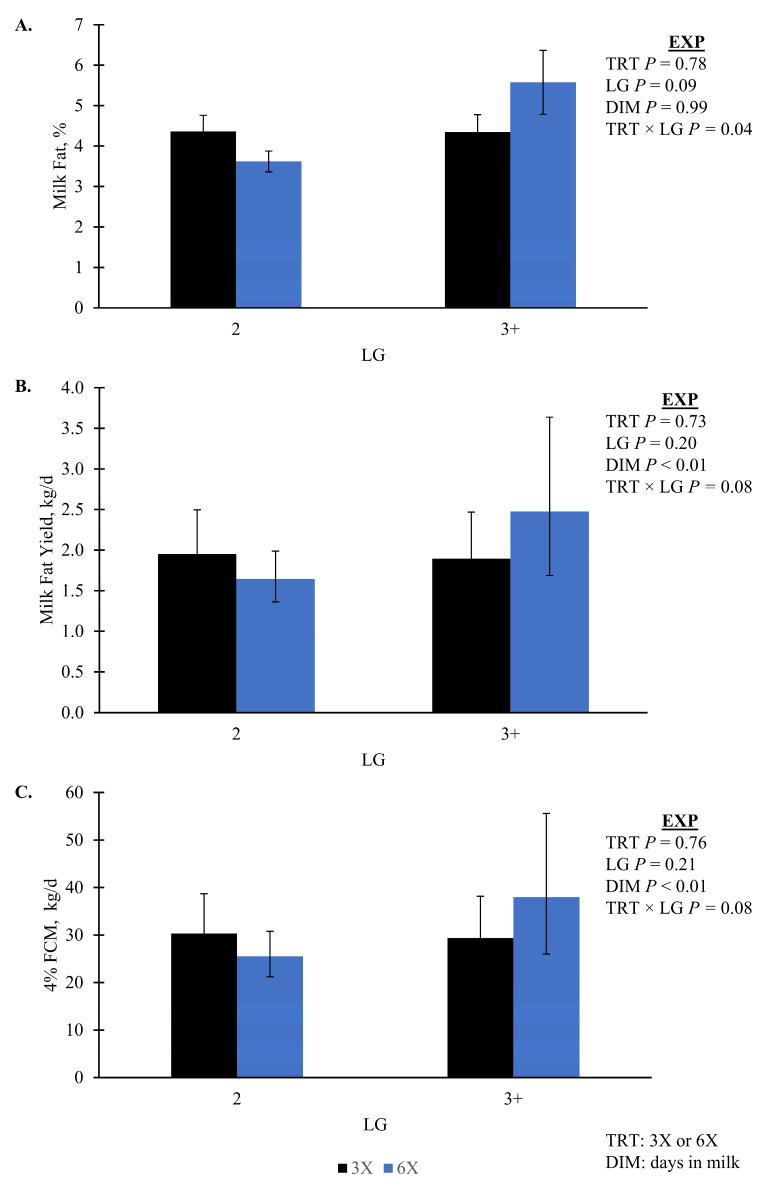
(**A**) Milk fat concentration, (**B**) yield, and (**C**) 4% fat corrected milk (FCM) for the experimental phase (EXP; 4 to 29 days in milk). Lactation groups are second parity (2 LG) and third or greater parity (3 + LG). Error bars represent the standard error of the means for panel (**A**), while panels (**B**,**C**) error bars represent the 95% confidence intervals of the means. Bars with different letters within the same panel indicate when there was significant evidence (*p* ≤ 0.05) of a difference.

Milk yield had marginal evidence of an interaction between TRT, LG, and DIM during the EXP (*p* = 0.09, TRT × LG × DIM); however, it was not separable by pairwise or simple effect comparisons. Milk yield during the EXP increased as lactation progressed for all groups (Figure 2A). During the CO, the 6X cows of 3 + LG had greater MY than 3X cows of the same lactation group, 62.9 ± 3.5 versus 53.2 ± 2.4 kg/d, respectively (simple effect *p* = 0.04, TRT × LG; Figure 2B). Milk protein yield had marginal evidence of an interaction effect of TRT, LG, and DIM during EXP (*p* = 0.07, TRT × LG × DIM). Daily milk fat yield, fat percentage, and FCM all had an interaction of TRT and LG that was not separatable between treatments while daily milk fat yield was greatest for 6X cows in 3 + LG (*p* ≤ 0.08; Figure 3). Milk components that did not differ by TRT (*p* > 0.10) included lactose %, protein %, solids not fat %, and milk urea nitrogen % (Table 3). Milk energy and ECM also did not differ by TRT (*p* > 0.10; Table 3).

Proportions of milk FA from different metabolic sources were altered by TRT, DIM, and interactions with LG (Table 4). Cows milked 3X compared to 6X had greater proportions of de novo FA, C4:0–C16:0 (51.70 ± 1.03 vs. 45.04 ± 1.63; *p* = 0.01) and odd- and branched-chain fatty acids (**OBCFA**; 2.26 ± 0.10 vs. 1.79 ± 0.15, *p* = 0.02; Table 4). The 3 + LG cows in 6X TRT had the lowest C6:0–C16:0, even-chain-saturated FA (*p* < 0.01; Table 4). These same cows had lower OBCFA, C13:0, *iso* C14:0, *iso* C15:0, *anteiso* C15:0, C15:0, and *iso* C16:0 (Table 4). Between the first and the fourth week of lactation, the OBCFA C13:0, *iso* C14:0, *iso* C15:0, *anteiso* C15:0, C15:0, increased (*p* ≤ 0.09). Opposingly, mono- and poly-unsaturated long-chain FA, C17:1, and greater increased in cows in the 6X group (*p* < 0.05; Table 4). An interaction between TRT × LG occurred, with the greatest proportion of this FA category observed in the 3+ group (*p* < 0.05; Table 4).

**Figure 4:**
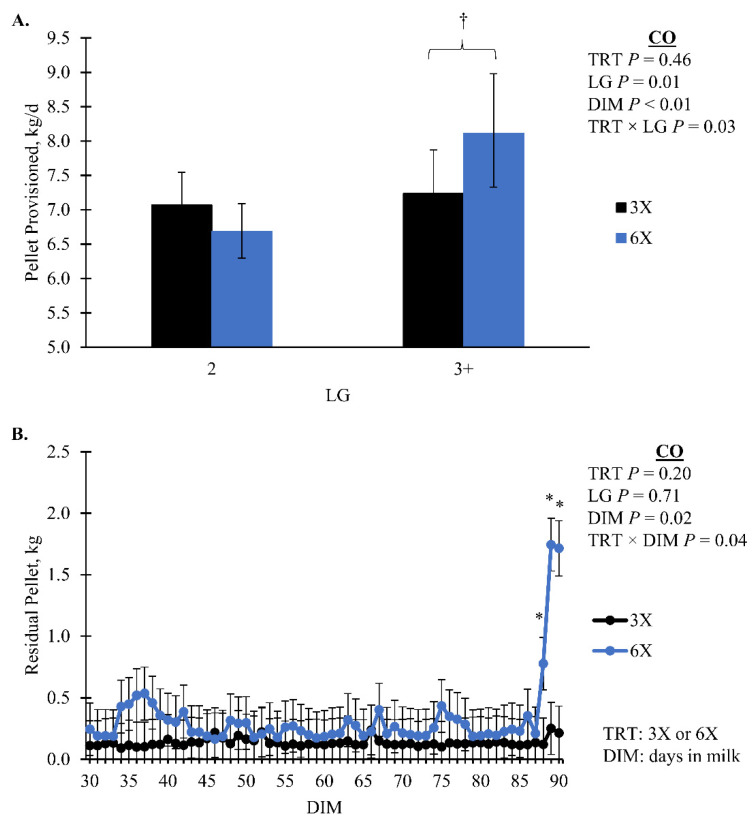
(**A**) Pellet provisioned and (**B**) residual pellet not dispensed for the carryover phase (CO; 30 to 90 days in milk, DIM). Lactation groups are second parity (2 LG) and third or greater parity (3 + LG). Error bars represent the 95% confidence intervals of the mean for panel (**A**) and the standard error of the means for panel (**B**). Simple effect comparisons of milking frequency treatment within the 3 + LG or DIM are indicated when there was significant (*; *p* ≤ 0.05) or marginal evidence (†; 0.05 < *p* ≤ 0.10) of a difference.

**Figure 5:**
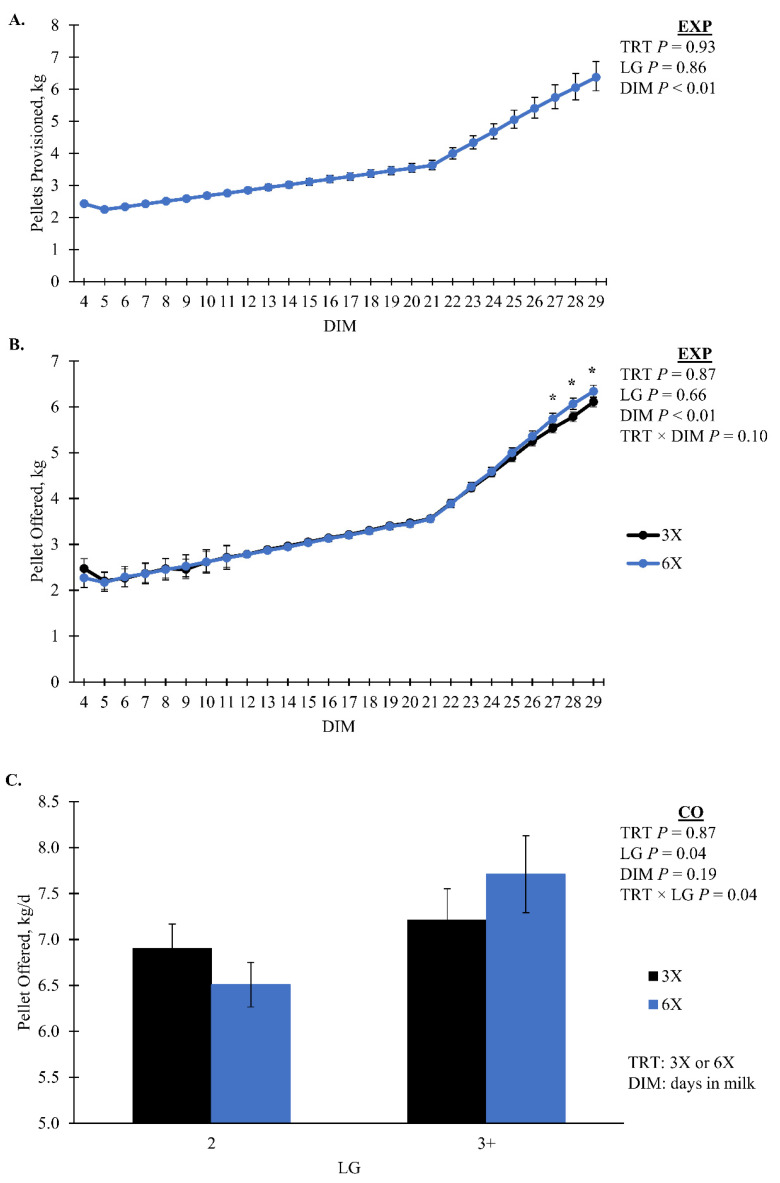
(**A**) Pellet provisioned and (**B**) offered during the experimental (EXP; 4 to 29 days in milk, DIM) as well as (**C**) pellet offered during the carryover phase (CO; 30 to 90 DIM). Lactation groups are second parity (2 LG) and third or greater parity (3 + LG). Error bars represent the standard error of the means. Simple effect comparisons of milking frequency treatment within DIM are indicated when there was significant evidence (*; *p* ≤ 0.05) of a difference.

**Figure 6:**
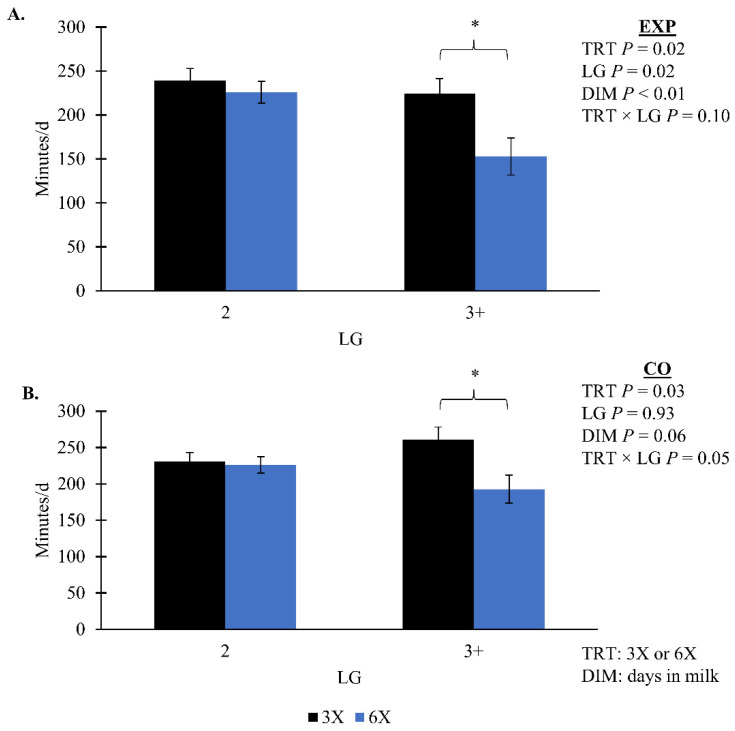
Total eating minutes per day during (**A**) the experimental (EXP; 4 to 29 days in milk) and (**B**) carryover phase (CO; 30 to 90 days in milk). Lactation groups are second parity (2 LG) and third or greater parity (3 + LG). Error bars represent the standard error of the means. Simple effect comparisons of milking frequency treatment within the 3 + LG indicate when there was significant evidence (*; *p* ≤ 0.05) of a difference.

Cows in the 6X TRT had a greater amount of residual pellet during the EXP compared to 3X cows, 0.08 [0.07, 0.09] and 0.06 [0.06, 0.07] kg/d, respectively (*p* < 0.01; Table 2). During the CO there was a TRT by DIM interaction where 6X had more residual pellets than 3X for the last three days (Figure 4B). As DIM increased during the EXP there was an increase in pellet offered (*p* = 0.10, TRT × DIM, Figure 5A). During the CO, the pellet offered had a TRT by LG interaction that was not separatable (*p* = 0.04; Figure 5B). Pellet provision was identical between TRT during EXP by design until 21 DIM when a milk yield-based feed table was implemented. The amount of pellet provision was greater for 6X cows in the 3 + LG, 8. 1 [7.3, 9.0] kg/d compared to 3X cows of the same LG 7.2 [6.7, 7.9] kg/d in the CO (simple effect *p* = 0.08; Figure 4A). During both phases, 3X cows in the 3 + LG had greater total eating minutes than 6X cows of the same LG (simple effect *p* = 0.02, TRT × LG; Figure 6). There was no TRT effect on the concentrate offered per 45 kg of milk or ECM during the EXP (*p* > 0.10). Rumination minutes did not differ by treatment for either phase (*p* > 0.10).

**Figure 7:**
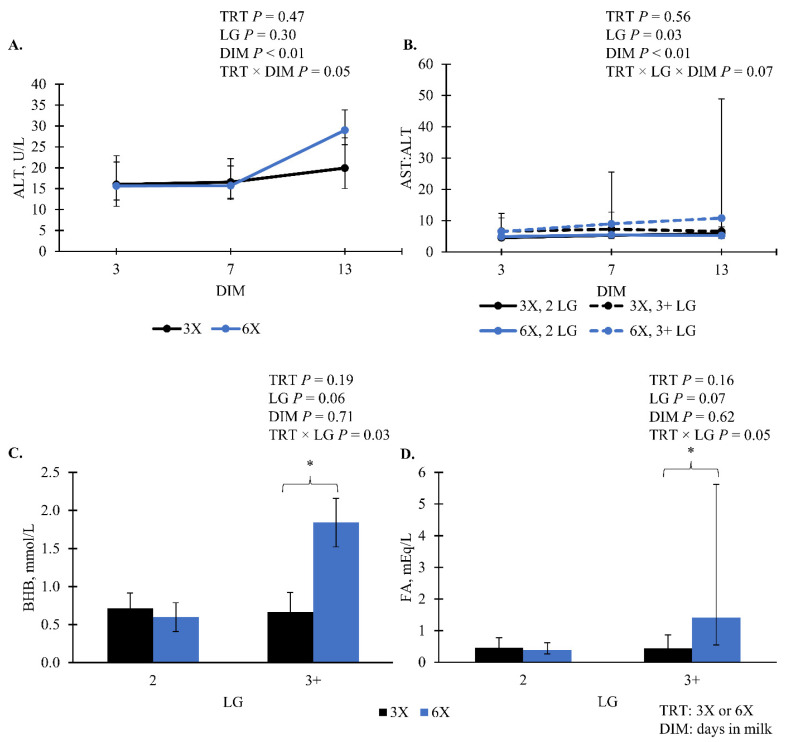
Blood plasma concentration of (**A**) alanine transaminase (ALT), (**B**) the ratio of aspartate transaminase (AST) to ALT (AST:ALT), (**C**) β-hydroxybutyrate (BHB), and (**D**) non-esterified fatty acids (FA) during the experimental phase (EXP, 4 to 29 days in milk). Lactation groups are second parity (2 LG) and third or greater parity (3 + LG). Error bars represent the 95% confidence interval of the means. Simple effect comparisons of milking frequency treatment within the 3 + LG or DIM are indicated when there was significant (*; *p* ≤ 0.05) evidence of a difference.

The following blood biomarkers did not have evidence of a TRT effect (*p* > 0.10; Table 5): albumin, AST, cholesterol, and glucose. There was an interaction of TRT and DIM effect for ALT, where ALT was greatest for 6X cows at 13 DIM compared to 3X cows and other DIM (*p* = 0.05; Figure 7A). For AST:ALT, there was marginal evidence of a 3-way interaction of TRT, LG, and DIM (*p* = 0.06; Figure 7B). Cows in the 6X, 3 + LG had greater AST:ALT as DIM increased compared to 3X, 3 + LG cows and 6X, 2 LG cows (Figure 7B). Within the 3 + LG, 6X cows had greater blood BHB 1.8 [0.8, 6.8] mmol/L (simple effect *p* = 0.04; Figure 7C) and FA 1.4 [0.6, 5.6] mEq/L (simple effect *p* = 0.05; Figure 7D) compared to 3X cows—0.7 [0.5, 1.1] mmol/L and 0.4 [0.3, 0.9] mEq/L, respectively.

**Figure 8:**
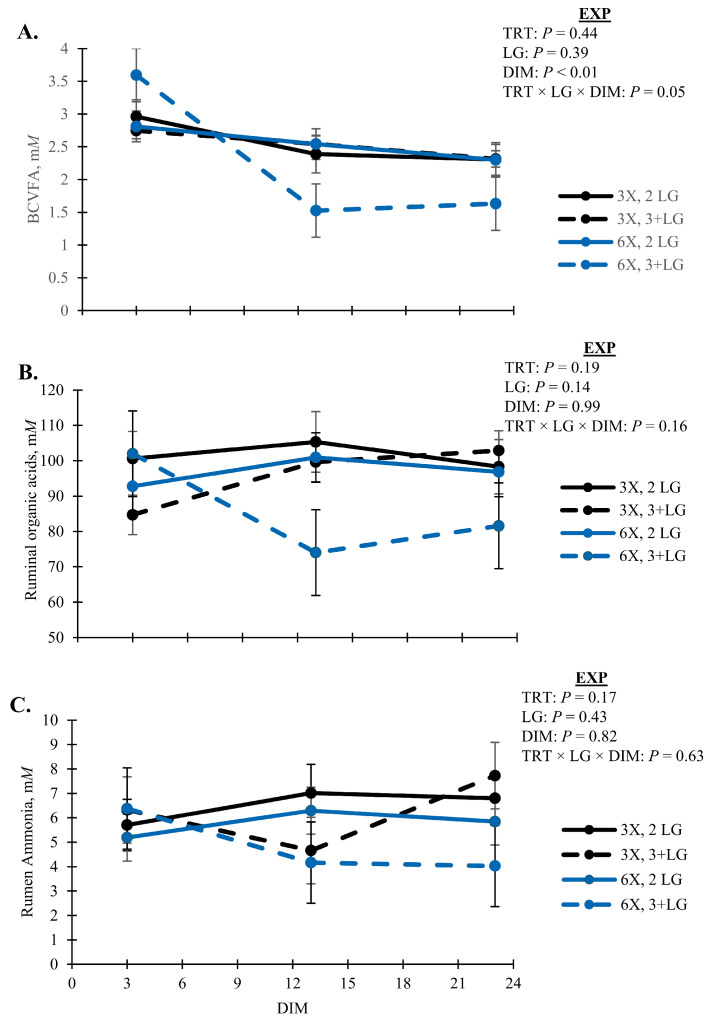
Rumen concentration of (**A**) branched-chain VFA (BCVFA), (**B**) total organic acids, and (**C**) ammonia during the experimental phase (EXP, 3 to 23 days in milk). Lactation groups are second parity (2 LG) and third or greater parity (3 + LG). Error bars represent the standard error of the means.

**Figure 9:**
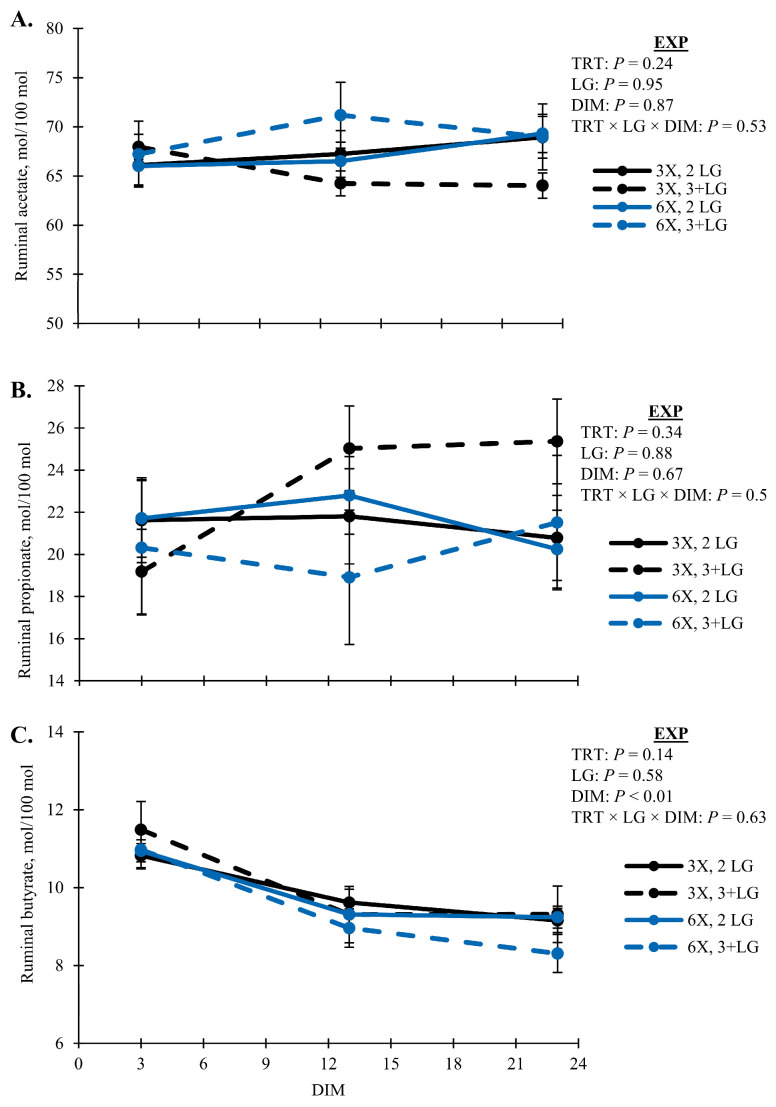
Rumen proportions of (**A**) acetate, (**B**) propionate, and (**C**) butyrate during the experimental phase (EXP, 3 to 23 days in milk). Lactation groups are second parity (2 LG) and third or greater parity (3 + LG). Error bars represent the standard error of the means.

Rumen fermentation profiles were unaffected by TRT and simple pairwise interactions between DIM and LG (*p* > 0.10; Table 6). Lactate was variable across animals in the EXP (Table 6). Concentrations of BCVFA decreased with DIM (*p* < 0.01) and was lowest for 6X cows in the 3 + LG during EXP (*p* = 0.05, TRT × LG × DIM; Figure 8A). Concentrations of total organic acids and ruminal ammonia followed a similar pattern as BCVFA (*p* > 0.10; Figure 8B,C). All cows had similar proportions of ruminal acetate (Figure 9A), and propionate (Figure 9B), except ruminal butyrate proportions decreased with DIM (*p* < 0.01) and was lowest for 3 + LG cows milked 6X (*p* = 0.05, TRT × DIM × LG; Figure 9C).

## 4. Discussion

In this study, we investigated the effect of MPD during early lactation when milked by an AMS in a controlled setting. To implement different milking frequencies, 16 cows were divided into two treatment groups to target an average number of MPD (3X) to a greater MPD (6X) [2]. Cows on the 6X treatment achieved an average of 5 ± 0.2 MPD, while the 3X TRT had an average of 3 ± 0.1 MPD. Exact MPD are challenging to enforce because cows still visit the AMS at voluntary times outside of the fetch schedule resulting in the AMS refusing cows with milking intervals shorter than the minimum. Additionally, AMS traffic can create extended ques that delay cows being milked voluntarily or after fetching. Nonetheless, our protocol did induce a significant difference in MPD between the treatment groups with the 6X group still representing a higher-than-normal MPD in early lactation.

The effects of greater MPD have been inconsistent in their magnitude between studies due to differences in MPD treatments and sampling methods, but overall, there is a consistent increase in milk yield with greater MPD [4,6,7,9,43,44]. Hale et al. [4] and Dahl et al. [5] investigated the effects of greater MPD and found that cows with four or six MPD, respectively, during early lactation (21 DIM) and lower MPD (two or three MPD, respectively) thereafter had carryover improvement to milk yield and components compared to the lower MPD controls. Although most milking frequency studies have reported positive effects of greater MPD during the early lactation [45], Fernandez et al. [46], VanBaale et al. [13], and Soberon et al. [43] reported results of minimal or negative carryover effects. Overall, reported TRT differences (greater MPD versus lower MPD) in carryover milk production ranged from −2.3 kg/d to +14 kg/d [4,5,7,13]. In the present study, greater milk production was observed for 3 + LG cows in the 6X TRT (+9.7 ± 4.1 kg/d) compared to 3 + LG cows in the 3X TRT during the CO. While the present study’s difference is based on a small number of cows, the milk yield difference was similar to the difference reported by Hanling et al. [7] and Perez-Hernandez et al. [44] when comparing two versus four MPD during early lactation. While the CO improvement in milk yield is consistent with previous work, the absence of a preceding improvement during the EXP was not. As we discuss subsequently, this may be a result of NEB that was promulgated in part by DIM based feed table.

Even with both LG consisting of multiparous cows, TRT by LG effects were often observed. In response to greater MPD, primiparous cows’ milk yield responses have been equal to or greater than those of multiparous cows [3,47,48]; however, others have reported the opposite [49,50]. Our study did not include primiparous cows due to the requirement of prior AMS training to encourage greater voluntarily milking visits. Between multiparous cows, Shoshani et al. [14] reported interactions of treatment and lactation where 3rd or greater lactation cows with six MPD did not differ from the three MPD cows of the same lactation, while cows in their 2nd lactation benefited from six MPD. As cows progress to their next lactation (i.e., 2nd to 3rd lactation), their potential milk production is expected to increase until plateauing at 4th or 5th lactation, leading to greater energy demands, mobilization of body reserves, and vulnerability to metabolic disorders [51]. Considering the differences in potential milk production by parity and resulting adaptations to meet this production, the 3 + LG cows likely experience a greater NEB during the EXP than 2 LG cows regardless of the MPD. This may help explain the current TRT × LG differences and is further discussed below.

Milk composition responses to varying MPD are also inconsistent. Evidence for TRT by LG differences were observed for milk fat yield and composition; however, mean differences did not separate. Numerically, we observed greater milk fat yield, composition, and FCM for 3 + LG, 6X cows during EXP suggests that these cows partitioned more energy towards milk fat production [22,52]. There was no difference in lactose, but milk protein yield had marginal evidence (*p* = 0.07, Figure 2C) of a 3-way interaction between TRT, LG, and DIM with no significant difference between treatment groups. Similarly, when comparing two and four MPD, cows milked four times a day produced more milk fat [53]. Hanling et al. [53] also reported an increase in milk protein for cows milked four times per day. In contrast, another study comparing two versus four MPD during the start of lactation observed decreased milk fat and protein for the four MPD group [4]. Wiking et al. [10] found that when cows in mid-to-late lactation were milked four times a day, there were no differences in fat content or percentage compared to cows milked twice a day. Part of the observed milk fat differences may be explained by the apparently greater FA mobilization for the 3 + LG, 6X cows, as demonstrated by the differences in BW and blood FA. In early lactation, these FA are a convenient source of endogenous FA that can be partitioned toward milk fat production [54]. The group with the greatest FA also had the greatest milk fat since around 40% of milk fat synthesis is from preformed FA [52,54].

Variations in the proportions of FA based on MPD and LG suggest different metabolic sources were being incorporated into milk fat. Cows in the 3 + LG were noticeably different when milked at 6X (Table 4). Typically, blood BHB is used as a precursor for de novo FA synthesis of short- and medium-chain FA in the mammary gland [55]. However, lipolysis during the early lactation from NEB results in greater incorporation of mono- and poly-unsaturated FA into milk triglycerides [22,56]. Greater proportions of long-chain FA in the 6X group and for 3+ cows suggest greater mobilization of adipose occurred to adapt to the energetic demands of greater MPD commonly observed in early lactation. Mammary epithelial gene expression in early lactation related to milk FA synthesis is decreased compared to later lactation, supporting the observed lower proportion of short- and medium-chain FA [57]. Additionally, increases in blood LCFA can downregulate the pathway for de novo synthesis and greater circulating FA in the 6X group could have occurred in this study [58]. Our results at 6X differ from previous research where first- and second-LG cows were determined to have greater mobilization of adipose reserves into milk triglycerides [59].

Fatty acids considered OBCFA in milk fat primarily arise from rumen microbial origin [60]. Cows milked 3X had greater proportions of OBCFA than 6X milked cows and increased over DIM. The 2 LG had greater proportions compared to 3 + LG (Table 4). Findings are consistent with previous studies where first or second lactation cows had greater proportions of OBCFA compared to 3 + LG [61,62]. The OBCFA were also lower in cows under 21 DIM compared to animals in later lactation [62], supporting increases in OBCFA by DIM in the current study. Although we cannot confirm in the current study, increases in OBCFA in 3X cows could be related to greater intake of those animals. Increases in both de novo and OBCFA support increases in VFA pools and contribution from microbial FA sources as lactation progresses and DMI increases. Additionally, the composition of the rumen microbiome is dependent on feed intake and could alter OBCFA profiles and VFA proportions for de novo milk FA synthesis [63].

A limitation to the present study was that individual cow PMR intake was not collected. Because of this, we cannot be certain of differences in cow energy or nutrient balance peripartum. Therefore, we have relied on indirect measures of energy status, like BW and blood chemistry, to indirectly infer cow metabolic status. Body weight had a 3-way interaction with TRT, LG, and wk where the average BW for cows in the 3 + LG was greater throughout both phases. However, BW change did not significantly differ between TRT in either study phases. On average, all cows except for the 6X, 3 + LG cows reached their starting BW by 90 DIM. Eslamizad et al. [6] reported that the 3X cows in their study began to gain weight before the groups milked more frequently. For both studies, the 3X cows likely entered a positive energy balance sooner than the 6X cows due to the lower demand of milk.

Blood biomarkers were examined to gain insight into shifts in nutrient usage and metabolic health in early lactation cows. Interestingly, the blood glucose concentrations in this study were not different between the two MPD implemented. This conflicts with findings from Eslmizad et al. [6] where the greater MPD groups, six MPD for the whole lactation and six MPD for the first 90 DIM, had lower blood glucose concentrations than the three MPD group. This difference in findings may be explained by the difference in lactose production in response to MPD where Eslmized et al. [6] reported greater lactose for 6X than 3X while the current study had no difference between treatments. The mammary gland uses blood glucose for lactose synthesis and with an increase in milk lactose content or milk yield, glucose will be drained from the blood circulation [64]. Similar to other studies testing different MPD [6,65], greater concentrations of FA and BHB were observed for 6X cows, but only those in the 3 + LG compared to 3X, 3 + LG cows. Elevated blood FA and BHB concentrations are signs that the cows may be in a NEB and are mobilizing adipose tissue for energy [19]. With both FA and BHB being elevated for 6X cows in the 3 + LG, it is possible that these cows were in a greater NEB and mobilizing adipose tissue to support milk production, especially for milk fat synthesis [22,54]. This is also supported by the previously discussed finding for milk FA where 6X, 3 + LG cows had greater proportions of long-chain milk FA that are preformed and can be from mobilized adipose compared to their 3X counterparts.

During times of elevated tissue mobilization and FA uptake by the liver, such as the transition period, liver cells can become damaged in the form of hepatic lesions [66]. The creation of these lesions results in the release of enzymes, including AST and ALT [66]. Increased levels of AST in blood serum are an indicator of liver damage, including damage that is considered subclinical [67]. It has also been reported that HYK cows have increased levels of AST compared to non-HYK cows [68]. Both AST and ALT have been studied in humans and are recognized as important indicators of liver health and damage [69]. In dairy cattle, AST, ALT, and the ratio of AST to ALT have been associated with liver TG and used to predict if cows have high liver TG status [70]. The ratio of AST to ALT had a 3-way interaction (*p* = 0.07, TRT × LG × DIM) shown in Figure 7B, where 6X, 3 + LG cows had the greatest ratio at 13 DIM. This finding is supported by the fact that this same group of cows also had greater concentrations of FA and BHB, indicating that they were likely mobilizing more adipose tissue and it was being taken up by the liver. Together, this data suggests that the 6X, 3 + LG cows had a metabolically stressed transition to lactation compared to their contemporaries.

Dry matter intake was greater for cows that had six MPD than three MPD in a previous study [6], while opposingly, McNamara et al. [65] reported that cows milked three times a day using a CMP did have a 1 kg decrease in feed intake compared to cows milked twice. It was hypothesized that the time needed for the extra milking away from the pen may have disrupted the feeding pattern for these cows [65]. Cows of the current study on 6X and in the 3 + LG had less total eating minutes compared to cows on 3X and in the same LG during both phases. We cannot confirm that less time spent eating meant the cows had less dry matter intake (**DMI**) without PMR intake data. However, blood biomarker responses (i.e., greater FA and BHB) and the greater preformed milk FA content observed for the 6X, 3 + LG cows are consistent with NEB. It is possible that the increased fetching frequency disrupted the time 6X cows had to eat during the EXP and could help explain their weight loss, delay in rebuilding body reserves, and blood biomarkers suggesting greater adipose mobilization as well as possible metabolic disfunction for 6X, 3 + LG cows.

Increasing feeding frequency and DMI in cows increases ruminal VFA concentrations and impacts the composition of the rumen microbiome [63,71]. The 3X milked cows with the greatest eating times also had the greatest ruminal concentrations of organic acids compared to 3 + LG cows with 6 MPD having the lowest concentrations. Rumen fermentation profiles support a potential reduction in DMI by the 6X cows, most noticeably the 3 + LG.

In addition to adipose reserves, ruminal butyrate would contribute to the elevated blood BHB observed in the 6X MPD cows in the 3 + LG to meet energy demands of lactation. Rumen butyrate proportions were lowest for the 3 + LG cows in the 6X group (Figure 9C). Preferential rumen epithelial uptake of VFA in order of butyrate > propionate > acetate has previously been reported [72]. Of this butyrate, an average of 49% was converted to ketone bodies by rumen epithelial metabolism based on observed differences in portal–arterial concentrations [73]. Other ketogenic VFA include the BCVFA, isobutyrate, and 3-methylbutyrate, which previously increased plasma BHBA when infused into fasted goats [74]. Metabolism by the rumen epithelium was reportedly similar to butyrate and conversion of ketone bodies [75]. In the current studies, 2- and 3-methylbutyrate co-eluded during VFA analysis. However, the total concentrations of BCVFA, including isobutyrate, were lowest for 6X MPD cows in the 3 + LG (Figure 8A).

Most studies looking at the effects of increased MPD used CMP to control the number of MPD [6,9,43]. While the use of a CMP has the benefit of consistent MPD and milking intervals, there is also an increase in labor needed to cover these additional milkings, cutting into the possible economic benefits of increasing MPD. An advantage to using an AMS is that milking frequency can be controlled to fit production level or lactation stage [50]. This advantage allows producers to make changes in milking frequency without the challenge of disrupting the flow of a milking parlor or needing to change the way cows are grouped in pens. The use of an AMS does open the number of milkings per day to variability, but there are methods that can be used to help motivate cows to visit the AMS more often.

Feeding palatable feedstuffs, such as pellets, serves multiple purposes, (1) enticing cows to voluntarily visit the AMS and (2) providing nutrients and energy [50,76]. While protocols and strategies vary, a feed table that scales concentrate provision to milk production is often used in an effort to better match nutrient supply and cow requirements [76]. Because 6X cows had potential for a milk production improvement, a controlled feed table with increases in pellet based on DIM was utilized. While controlling for a difference in pellet provision, this strategy is used in applied practice to incrementally adapt the rumen environment and prevent acidosis [31,77]. As previously stated, the controlled pellet provision during EXP for the first 21 DIM may have prohibited 6X cows from achieving the expected milk production improvement. The implementation of the milk-based feed table earlier may have alleviated some of their NEB. Additionally, residual pellet intake also demonstrates that 6X cows were not accessing their entire allotment, possibly exacerbating their NEB. Indications of NEB (i.e., blood chemistry) and partitioning energy towards milk fat are evidence of a dietary limitation in the 6X, 3 + LG cows. Additionally, the CO milk production improvement for 6X, 3 + LG cows was concomitant with the transition to a milk-based feed table. In theory, this transition may have improved nutrient supply to 6X, 3 + LG cows; however, other research has demonstrated that greater pellet provision in AMS may reduce PMR intake [78,79,80] and increase daily variation in pellet offered [79,80,81]. Tatone et al. [29] reported that between CMP and AMS herds, AMS herds had 5% greater prevalence of high concentrations of milk BHB than CMP herds, while others have shown no difference [82,83,84]. This difference in high milk BHB prevalence could be due to some farms not feeding enough energy through pellet and PMR. This was seen in the current study, in which 6X cows may have been limited on energy intake with pellet provisioned during the EXP. The amount of pellet feed a cow can eat during a day is dependent on MPD, time in the AMS, dispensing rate of pellets, and the rate that cows can eat [76,80]. With more MPD, a larger amount of pellet feed can be provisioned per day because the pellets are divided over more milkings. Overall, future research on AMS feed table strategies should consider establishing recommendations for pellet provision based on MPD or milk yield in early lactation to promote milk productivity and lessen indicators of metabolic illness.

## 5. Conclusions

The present study adds to the previous literature investigating the effects of milking frequency. By using an AMS instead of a CMP, there was more opportunity for variation in milking intervals and MPD. Unlike previous studies on MPD, cows with an increased MPD did not have a clear increase in milk production during the EXP, however consistent with previous studies, cows milked with increased MPD did have an increase in milk production during the CO after the MPD was reduced. Many of the significant treatment effects observed in the current study had an interaction with LG. Cows in the 6X treatment and of 3 + LG had responses suggesting they experienced a greater NEB during the EXP than their 3X contemporaries, including greater blood FA, BHB, and AST to ALT ratio at 13 DIM, greater preformed milk FA content, as well as reduced levels of ruminal butyrate and BCVFA. The mechanism of the more severe NEB may be due to limited nutrient intake from 4 to 21 DIM as a result of a DIM-based feed table and reduced time spent eating. These data suggest value in future research exploring milking and nutrition-based management strategies employed by AMS that balance pellet feeding and MPD in a parity-dependent manner.

## Figures and Tables

**Table 1:** Diet composition of feed ingredients and proximate analysis ^1^.

Diet Variables	Inclusion, % DM
Feed Ingredients	
Corn silage	33.7
Alfalfa silage	11.5
Baleage	12.7
Concentrate ^2^	15.4
Corn, ground	14.3
MS pellet ^3^	11.8
Mineral ^4^	0.5
Analysis ^5^	
DM	50.3
CP	16.41
aNDFom	27
NFC	43.76
WSC	3.41
Starch	31.77
EE	3.8
Ash	9.03
NEL3×, Mcal/kg DM	1.72

^1^ Partial mixed ration feed intake was not measured. During formulation, total dry matter (DM) intake was assumed to be 60 kg/d. Diet energy content was based on CNCPS v. 6.55. ^2^ Ingredients (% DM of mix, >1% inclusion): ground corn (25.7%), extruded soybean meal (22.1%, AminoPlus, AGP, Dawson, MN, USA), expellers soybean meal (13.3%), SQ-810 (4.8%, Arm & Hammer), palm fat (4.0%), meat and bone meal (3.3%, pork), metaatein (2.3%), calcium salts of fatty acids (2.4%), calcium carbonate (2.2%), and sodium chloride (1.6%). ^3^ Automated milking system (AMS) pellet inclusion set to base rate of 3.6 kg/d, as fed (3.1 kg/d, DM). Ingredients (% DM of pellet, >1% inclusion): ground corn (18.1%), wheat middlings (16.8%), soybean hulls (13.2%), wheat (13.3%, red dog), extruded soybean meal (8.7%, AminoPlus, AGP), corn starch (3.7%), molasses (1.6%), calcium carbonate (2.0%). ^4^ Mixture was composed of 33.3% Immuno-Plus Dairy, 30.3% monocalcium phosphate, 27.3% DYNAMATE (Mosaic, Tampa, FL, USA), and 9.1% yeast culture (Cellerate HD, Phibro Teaneck, NJ, USA). ^5^ CP = crude protein, aNDFom = neutral detergent fiber organic matter, NFC = nonfibrous carbohydrate, WSC = water soluble carbohydrate, EE = ether extract.

**Table 2:** Parity, milkings per day (MPD), body weight (BW) variables, pellet variables, and rumination minutes for cows milked 3 or 6 times a day (3X or 6X) ^1^.

		Treatments				*p*-Values ^2^				
		3X		6X		TRT	TRT × LG	TRT × DIM	LG × DIM	TRT × LG × DIM
Variables ^3^	Phase	LSM	VAR	LSM	VAR					
Parity		2.31	0.26	2.37	0.26	0.85	-	-	-	-
MPD										
	EXP	3.10	0.14	4.96	0.19	<0.01	0.75	0.99	0.99	-
	CO	3.65	0.23	3.59	0.23	0.82	0.43	1	1	-
BW, kg										
	EXP	675.46	17.48	697.91	19.55	0.37	0.35	0.03	0.57	0.05
	CO	676.82	14.77	687.60	16.51	0.64	0.88	<0.01	<0.01	<0.01
BW Change, kg										
	EXP	−16.68	11.10	−79.39	67.79	0.83	0.43	-	-	-
	CO	24.66	14.82	−45.37	35.34	0.11	0.30	-	-	-
Pellet Provisioned, kg/d										
	EXP	3.29	[3.25, 3.32]	3.29	[3.25, 3.34]	0.93	0.36	1	1	-
	CO	7.16	[6.78, 7.55]	7.36	[6.94, 7.81]	0.46	0.03	0.37	0.95	-
Residual Pellet, kg/d										
	EXP	0.06	[0.06, 0.07]	0.08	[0.07, 0.09]	<0.01	0.84	0.32	0.54	0.23
	CO	0.14	0.11	0.32	0.11	0.20	0.35	0.04	<0.01	-
Pellet Offered, kg/d										
	EXP	3.36	[3.32, 3.40]	3.37	[3.17, 3.41]	0.87	0.42	0.10	0.84	0.98
	CO	7.06	0.22	7.11	0.24	0.87	0.04	0.27	0.06	-
Total Eating Minutes, min/d										
	EXP	231.78	10.95	189.33	12.25	0.02	0.10	0.81	0.67	0.27
	CO	245.84	10.38	209.38	11.10	0.03	0.05	0.91	0.25	0.15
Concentrate, kg/45 kg milk										
	EXP	3.30	[3.12, 3.51]	3.29	[3.06, 3.55]	0.92	0.59	0.33	0.17	0.66
	CO	3.59	0.10	3.48	0.13	0.74	0.35	0.22	0.95	-
Concentrate kg/45 kg ECM										
	EXP	0.07	0.01	0.07	0.01	0.55	0.35	0.85	0.10	-
Rumination Minutes, min/d										
	EXP	5633.20	[5244.66, 5996.61]	5873.08	[5499.27, 6224.48]	0.29	0.60	0.70	0.93	0.12
	CO	56,950.50	[54,439.10, 59,257.90]	59,690.80	[57,141.30, 62,039.30]	0.11	0.60	0.18	0.02	-

^1^ Data are presented as least squares means (LSM) and their variation (VAR). Variation is the standard error of the mean for response variables not transformed prior to mixed model analysis, while transformed variables had the 95% confidence interval [lower limit, upper limit] of the LSM reported. ^2^ Experimental (EXP; 3 to 29 days in milk, DIM) and carryover (CO; 30 to 90 DIM) phases were used. Cows across treatments (TRT) were in lactation groups of second parity (2 LG) and third or greater parity (3 + LG). All interactions were included and then backwards eliminated based on if model Bayes information criterion improved. ^3^ The following are the definitions of variables included in the table. Body weight (BW) was calculated using a rolling 3-day average while BW change was calculated as the difference from the BW at the start of each phase to the last day of each phase. All interactions with DIM for the variables BW are by wk instead of DIM. Pellet provisioned was the total amount of pellet feed that a cow could be offered in a day. Residual pellet was the amount of pellet that was not dispensed to the cow from the total amount of pellet available. Pellet offered was the amount of pellet feed that was offered to the cow. Concentrate/45 kg milk is the amount of pellet feed per 45 kg of milk. Concentrate/45 kg milk is the amount of pellet feed per 45 kg of energy-corrected milk (ECM; Tyrrell and Reid. 1965, 0.327 × Milk, kg + 12.95 × Milk Fat, kg + 7.2 × Milk CP, kg).

**Table 3:** Milk production and milk sample composition for cows milked 3 or 6 times per day (3X or 6X) ^1^.

	Treatment				*p*-Value ^2^				
	3X		6X		TRT	TRT × LG	TRT × DIM	LG × DIM	TRT × LG × DIM
Variables ^3^	LSM	VAR	LSM	VAR					
Milk Yield, kg/d									
EXP	44.41	1.54	46.47	1.94	0.45	0.79	0.02	0.28	0.09
CO	53.08	1.57	55.78	1.98	0.34	0.01	0.80	0.68	-
Milk Components									
Fat, kg/d	1.08	[1.61, 2.30]	1.10	[1.62, 2.51]	0.73	0.08	0.82	0.91	-
Protein, kg/d	1.37	[1.22, 1.57]	1.27	[1.12, 1.47]	0.39	0.60	0.24	0.26	0.07
Lactose, kg/d	1.99	[1.76, 2.27]	1.92	[1.66, 2.25]	0.72	0.46	0.83	0.07	-
Fat, %	4.4	0.29	4.5	0.37	0.78	0.04	0.99	0.53	-
Protein, %	3.20	0.14	2.86	0.16	0.37	0.87	0.41	<0.01	-
Lactose, %	4.67	0.20	4.28	0.23	0.45	0.62	0.75	0.13	-
SNF, %	9.03	0.38	8.13	0.42	0.18	0.52	0.61	0.04	-
MUN, mg/dL	10.84	2.80	10.28	3.28	0.92	0.94	1	1	-
Calculated milk energy									
ECM, kg/d	49.59	[43.51, 56.51]	50.91	[43.39, 59.73]	0.80	0.19	0.60	0.59	-
4% FCM, kg/d	29.85	[25.03. 35.03]	31.16	[25.13, 38.64]	0.76	0.08	0.80	0.90	-
Milk Energy, Mcal/d	0.79	[0.70, 0.89]	0.74	[0.64, 0.86]	0.54	0.19	0.88	0.71	-

^1^ Data are presented as least squares means (LSM) and their variation (VAR). Variation is the standard error of the mean for response variables not transformed prior to mixed model analysis, while transformed variables had the 95% confidence interval [lower limit, upper limit] of the LSM reported. ^2^ Statistics were run separately for the experimental (EXP; 3 to 29 days in milk, DIM) and carryover (CO; 30 to 90 DIM) phases. Cows across treatments (TRT) were in lactation groups of second parity (2 LG) and third or greater parity (3 + LG). The TRT × LG × DIM interaction was backwards eliminated if model Bayes information criterion improved. ^3^ The following are the definitions of variables included in the table: solids not fat (SNF), milk urea nitrogen (MUN), energy-corrected milk (ECM; Tyrrell and Reid, 1965, 0.327 × Milk, kg + 12.95 × Milk Fat, kg + 7.2 × Milk CP, kg), 4% fat-corrected milk (4% FCM; Gains and Davidson, 1923, 0.4 × Milk, kg + 15 × Milk Fat, kg), and daily milk energy (NRC, 2001, 9.29 × Fat, kg/Milk, kg + 5.85 × TP, kg/Milk, kg + 3.95 × Lactose, kg/Milk, kg).

**Table 4:** Milk fatty acid proportions (g/100 g of FAME) from cows milked 3 or 6 times (3X or 6X) in second or third and greater parity (2 LG or 3 + LG) ^1^.

		Treatment				*p*-Value ^2^				
		3X		6X		TRT	LG	DIM	TRT × LG	LG × DIM
Variables	Lactation Group	LSM	VAR	LSM	VAR					
C4:0	2	3.31	0.07	3.20	0.06	0.70	0.69	0.03	0.22	0.80
	3+	3.08	0.14	3.30	0.23					
C6:0	2	2.00	0.06	1.97	0.06	0.07	0.51	0.11	0.07	0.81
	3+	2.14	0.13	1.63	0.22					
C8:0	2	1.09	0.05	1.09	0.05	0.01	0.31	0.02	0.01	0.65
	3+	1.27	0.10	0.67	0.17					
C10:0	2	1.98	0.11	2.06	0.10	<0.01	0.30	<0.01	<0.01	0.60
	3+	2.57	0.23	0.89	0.39					
C11:0	2	0.15	0.01	0.17	0.01	0.03	0.30	<0.01	<0.01	0.80
	3+	0.20	0.03	0.05	0.05					
C12:0	2	2.05	0.11	2.14	0.10	<0.01	0.23	<0.01	<0.01	0.12
	3+	2.74	0.24	0.78	0.39					
C13:0	2	0.08	0.01	0.09	0.01	0.02	0.20	0.01	<0.01	0.25
	3+	0.12	0.02	-	0.03					
*Iso* C14:0	2	0.73	0.05	0.84	0.04	0.32	0.09	0.11	0.04	0.47
	3+	0.74	0.09	0.44	0.16					
C14:0	2	8.09	0.26	8.14	0.24	<0.01	0.62	0.01	<0.01	0.20
	3+	10.08	0.55	5.54	0.91					
*Iso* C15:0	2	0.136	0.004	0.136	0.004	<0.01	0.07	0.06	<0.01	0.10
	3+	0.144	0.009	0.087	0.015					
*Anteiso* C15:0	2	0.31	0.01	0.30	0.01	0.01	0.08	0.06	0.02	0.38
	3+	0.32	0.02	0.20	0.03					
C14:1 *cis*-9	2	0.55	0.02	0.61	0.02	0.97	0.49	<0.01	0.18	0.17
	3+	0.57	0.05	0.50	0.08					
C15:0	2	0.74	0.04	0.78	0.04	0.04	0.06	0.03	0.01	0.28
	3+	0.78	0.08	0.36	0.13					
*Iso* C16:0	2	0.22	0.01	0.22	0.01	0.03	0.26	0.45	0.02	0.36
	3+	0.24	0.02	0.15	0.03					
C16:0	2	30.44	0.43	30.96	0.40	0.03	0.58	0.21	<0.01	0.71
	3+	32.55	0.90	27.71	1.49					
*Iso* C17:0	2	0.35	0.01	0.34	0.01	0.66	0.04	0.90	0.90	0.27
	3+	0.30	0.02	0.29	0.03					
*Anteiso* C17:0	2	0.60	0.02	0.60	0.01	0.69	0.02	0.72	0.75	0.16
	3+	0.49	0.03	0.51	0.05					
C16:1 *cis*-9	2	2.13	0.10	2.16	0.09	0.04	0.99	0.83	0.04	0.98
	3+	1.70	0.20	2.60	0.33					
C17:0	2	0.76	0.02	0.78	0.02	0.49	0.09	0.17	0.93	0.24
	3+	0.67	0.04	0.70	0.07					
C17:1 *cis*-10	2	0.32	0.01	0.34	0.01	0.02	0.84	0.28	0.03	0.75
	3+	0.24	0.03	0.40	0.05					
C18:0	2	12.23	0.29	12.29	0.27	0.39	0.84	<0.01	0.42	0.24
	3+	11.90	0.61	12.91	1.01					
C18:1 *cis*-9	2	25.87	0.95	24.76	0.87	0.02	0.22	0.15	<0.01	0.39
	3+	22.06	1.97	34.44	3.28					
C18:1 *cis*-11	2	0.31	0.02	0.33	0.02	0.38	0.02	0.54	<0.01	0.39
	3+	0.24	0.04	0.15	0.07					
C18:1 total	2	0.05	0.03	0.09	0.03	0.85	0.64	0.69	0.38	0.42
	3+	0.07	0.06	0.01	0.10					
C19:0	2	0.16	0.01	0.17	0.01	<0.01	0.94	0.61	0.04	0.37
	3+	0.13	0.01	0.19	0.02					
C18:2 *cis*-9, *cis*-12	2	2.48	0.06	2.55	0.06	0.07	0.96	0.16	0.14	0.63
	3+	2.29	0.13	2.73	0.21					
C20:1 *cis*-11	2	0.061	0.005	0.059	0.005	0.82	0.38	0.05	0.98	0.32
	3+	0.051	0.010	0.049	0.017					
C18:3 *cis*-9, *cis*-12, *cis*-15	2	0.38	0.03	0.39	0.02	0.24	0.31	<0.01	0.22	0.07
	3+	0.26	0.05	0.39	0.09					
CLA *cis*-9, *trans*-11	2	0.30	0.01	0.30	0.01	0.27	0.22	0.40	0.49	0.56
	3+	0.26	0.02	0.29	0.03					
C20:3n6	2	0.010	0.005	0.024	0.004	0.57	0.55	0.99	0.36	0.34
	3+	0.012	0.010	0.009	0.016					
C20:4n6	2	0.15	0.01	0.18	0.01	0.19	0.14	0.05	0.97	0.70
	3+	0.12	0.02	0.15	0.04					
SCFA ^3^	2	48.96	0.90	49.56	0.83	<0.01	0.41	0.03	<0.01	0.35
	3+	54.44	1.86	40.52	3.10					
OBCFA ^4^	2	2.36	0.09	2.53	0.09	0.02	0.03	0.01	<0.01	0.20
	3+	2.53	0.19	1.28	0.32					
LCFA ^5^	2	43.59	0.93	42.66	0.85	<0.01	0.25	0.02	<0.01	0.24
	3+	38.73	1.93	52.73	3.21					
Total Unknowns (C6:0–C22:6)	2	0.09	0.01	0.12	0.01	0.84	0.71	0.33	0.36	0.14
	3+	0.10	0.03	0.08	0.05					

^1^ Data are presented as least squares means (LSM) and their variation (VAR). Variation is the standard error of the mean for response variables not transformed prior to mixed model analysis. ^2^ Samples were grouped by 3–8 and 23–28 DIM. Cows across treatments (TRT) were in lactation groups of second parity (2 LG) and third or greater parity (3 + LG). ^3^ Sum of saturated, even-chained fatty acids C4:0–C16:0. ^4^ Sum of odd- and branched-chain fatty acids C11:0–*iso* C16:0. ^5^ Sum of long-chain fatty acids C18:0 and greater, including all known fatty acids.

**Table 5:** Least squares means (LSM) and variance (VAR) of blood analytes for cows milked 3 or 6 times per day (3X or 6X) from the experimental phase (EXP; 3 to 29 days in milk, DIM).

	Treatments				*p*-Value				
	3X		6X		TRT	TRT × LG	TRT × DIM ^2^	LG × DIM ^2^	TRT × LG × DIM ^2^
Biomarkers ^1^	LSM	VAR ^3^	LSM	VAR					
Albumin, g/dL	3.33	0.18	3.40	0.19	0.74	0.44	0.81	0.48	0.31
ALT, U/L	17.40	[12.66, 24.85]	18.99	[14.63, 25.29]	0.47	0.31	0.05	0.65	-
AST, U/L	101.92	[82.17, 134.19]	110.89	[85.69, 156.99]	0.63	0.28	0.48	0.68	-
AST:ALT	5.90	[4.89, 7.44]	6.42	[5.12, 8.59]	0.58	0.58	0.76	0.99	0.06
BHB, mmol/L	0.69	[0.55, 0.92]	0.90	[0.66, 1.42]	0.19	0.03	0.34	0.87	0.72
Cholesterol, mg/dL	59.88	[45.62, 78.61]	63.65	[48.53, 83.50]	0.53	0.19	0.38	0.64	-
Glucose, mg/L	5770.90	[5296.35, 6178.09]	5789.66	[5255.16, 6240.25]	0.95	0.48	0.21	0.85	0.16
FA, mEq/L	0.45	[0.31, 0.68]	0.69	[0.43, 1.20]	0.16	0.06	0.49	0.19	-

^1^ The following are the definitions for the biomarker abbreviations: alanine transaminase (ALT), aspartate transaminase (AST), ratio of AST to ALT (AST:ALT), β-hydroxybutyrate (BHB), and fatty acids (FA). ^2^ Samples were grouped by DIM as 3, 8, and 13. ^3^ Variation includes standardized error of the mean or 95% confidence intervals. Confidence intervals were used when variables were transformed for normality.

**Table 6:** Average rumen fermentation responses for cows milked 3 or 6 times per day (3X or 6X) ^1^.

	Treatment				*p*-Value ^2^				
	3X		6X		TRT	TRT × LG	TRT × DIM	LG × DIM	TRT × LG × DIM
Variables	LSM	VAR	LSM	VAR					
Organic Acid ^3^, mM	99	4	91	4	0.19	0.63	0.30	0.59	0.16
Acetate, mol/100 mol	66.41	1.02	68.20	1.12	0.24	0.59	0.49	0.49	0.53
Propionate, mol/100 mol	22.30	0.97	20.91	1.06	0.34	0.28	0.62	0.38	0.52
Butyrate, mol/100 mol	9.95	0.15	9.62	0.16	0.14	0.19	0.88	0.33	0.63
Valerate, mol/100 mol	1.34	0.06	1.26	0.06	0.33	0.42	0.53	0.38	0.17
Lactate, mM	1.37	[0.12, 15.5]	3.16	[1.22, 8.13]	0.54	0.19	0.81	0.81	-
Ammonia, mM	6.37	0.50	5.32	0.55	0.17	0.67	0.48	0.25	0.63
Free AA, mM	4.30	0.68	4.53	0.75	0.82	0.17	0.52	0.93	0.54
BCVFA ^4^, mM	2.5	0.1	2.4	0.1	0.44	0.45	0.18	0.24	0.05

^1^ Data are presented as least squares means (LSM) and their variation (VAR). Variation is the standard error of the mean for response variables not transformed prior to mixed model analysis, while transformed variables had the 95% confidence interval [lower limit, upper limit] of the LSM reported. ^2^ Samples were grouped as 3, 13, and 23 DIM. Cows across treatments (TRT) were in lactation groups of second parity (2 LG) and third or greater parity (3 + LG). The TRT × LG × DIM interaction was backwards eliminated if Bayes information criteria improved. ^3^ Sum of acetate, propionate, butyrate, valerate, and lactate. ^4^ BCVFA = branched-chain VFA, sum of isovalerate, 2-methybutyrate, and isobutyrate.

## Data Availability

The original contributions presented in the study are included in the article/Supplementary Materials. Further inquiries can be directed to the corresponding author.

## Supplementary Materials

The following supporting information can be downloaded at: https://www.mdpi.com/article/10.3390/ani14162293/s1, Table S1: Inter- and median intra-assay coefficients of variation (CV) for quantified analytes; Table S2: Response variable transformation and final linear mixed models.
