# Supplementary material for: Effects of Early Lactation Milking Frequency in an Automated Milking System on Cow Performance
Source: Animals (Basel). 2024 Aug 6;14(16):2293. doi: 10.3390/ani14162293 (PMC11350756; doi:10.3390/ani14162293)
Supplement: Supplementary file 1 [file animals-14-02293-s001.zip › milkingFrequency_animalsDraft_supplement.pdf]

Table S1. Inter- and median intra-assay coefficients of variation (CV) for quantified analytes.

Analytes	Inter-assay CV, %	Intra-assay CV, %
Albumin	1.28	1.66
Alanine transaminase	9.58	3.85
Aspartate transferase	8.36	0.00
β-hydroxybutyrate	0.00	0.06
Cholesterol	6.87	1.66
Glucose	1.22	1.21
Non-esterified fatty acids	2.29	0.77

Table S2. Response variable transformation and final linear mixed models

Response			Model Parameters[4]									
Variable[1]	f(x)[2]	Phase[3]	Dist.	Link	COV[5]	TRT	TRT × LG	TRT × DIM	LG × DIM	3-way	VCOV[6]
Parity	-		Pois	Log	-	0.85	-	-	-	-	-
MPD	-	EXP	Pois	Log	-	<0.01	0.75	0.99	0.99	-	-
	-	CO	Pois	Log	-	0.82	0.43	1	1	-	-
BW, kg	-	EXP	Nor	Id	-	0.37	0.35	0.03	0.57	0.05	VC
	-	CO	Nor	Id	-	0.64	0.88	<0.01	<0.01	<0.01	-
BW Change, kg	-	EXP	Nor	Id	-	0.83	0.43	-	-	-	-
	-	CO	Nor	Id	-	0.11	0.30	-	-	-	-
Pellet Provisioned, kg/d	$1/x$	EXP	Nor	Id	-						VC
	Log10(x)	CO	Nor	Id	-	0.46	0.03	0.37	0.95	-	ar(1)
Residual Pellet, kg/d	$1/x^{0.5}$	EXP	Nor	Id		<0.01	0.84	0.32	0.54	0.23	-
	-	CO	Nor	Id	-	0.20	0.35	0.04	<0.01	-	ar(1)
Pellet Offered, kg/d	-	EXP	Nor	Id	-	0.87	0.42	0.10	0.84	0.98	-
	-	CO	Nor	Id	-	0.87	0.04	0.27	0.06	-	ar(1)
TEM, min/d	-	EXP	Nor	Id	-	0.02	0.10	0.81	0.67	0.27	ar(1)
	-	CO	Nor	Id	-	0.03	0.05	0.91	0.25	0.15	-
Con., kg/45 kg milk	$1/x$	EXP	Nor	Id	PME305	0.92	0.59	0.33	0.17	0.66	arh(1)
	-	CO	Nor	Id	PME305	0.74	0.35	0.22	0.95	-	ar(1)
Con., kg/45 kg ECM	-	EXP	Beta	Logit	PME305	0.55	0.35	0.85	0.10	-	-
Rumination Min, min/d	$x^{2/100}$	EXP	Nor	Id	-	0.29	0.60	0.70	0.93	0.12	-
	$x^{3/1000}$	CO	Nor	Id	-	0.11	0.60	0.18	0.02	-	ar(1)
MY, kg/d	-	EXP	Nor	Id	PME305	0.45	0.79	0.02	0.28	0.09	arh(1)
	-	CO	Nor	Id	PME305	0.34	0.01	0.80	0.68	-	arh(1)
Fat, kg/d	Log10(x)	EXP	Nor	Id	PME305	0.73	0.08	0.82	0.91	-	ar(1)
Protein, kg/d	$1/x$	EXP	Nor	Id	PME305	0.39	0.60	0.24	0.26	0.07	ar(1)
Lactose, kg/d	$1/x^{0.5}$	EXP	Nor	Id	PME305	0.72	0.46	0.83	0.07	-	-
Fat, %	$x/100$	EXP	Beta	Logit	PME305	0.78	0.04	0.99	0.53	-	-
Protein, %	$x/100$	EXP	Beta	Logit	PME305	0.37	0.87	0.41	<0.01	-	-
Lactose, %	$x/100$	EXP	Beta	Logit	PME305	0.45	0.62	0.75	0.13	-	-
SNF, %	$x/100$	EXP	Beta	Logit	PME305	0.18	0.52	0.61	0.04	-	-
MUN, mg/dL	$x/100$	EXP	NB	logit	PME305	0.92	0.94	1	1	-	-
ECM, kg/d	Log10(x)	EXP	Nor	Id	PME305	0.80	0.19	0.60	0.59	-	ar(1)
4% FCM, kg/d	Log10(x)	EXP	Nor	Id	PME305	0.76	0.08	0.80	0.90	-	ar(1)
Milk Energy, Mcal/d	Log10(x)	EXP	Nor	Id	PME305	0.54	0.19	0.88	0.71	-	ar(1)
ALT, U/L	$1/x^{1/3}$	EXP	Nor	Id	-	0.47	0.31	0.05	0.65	-	-
AST, U/L	$1/x$	EXP	Nor	Id	-	0.63	0.28	0.48	0.68	-	-
AST:ALT	$1/x$	EXP	Nor	Id	-	0.58	0.58	0.99	0.76	0.06	-
BHB, mmol/L	$1/x$	EXP	Nor	Id	-	0.19	0.03	0.34	0.87	0.72	-
Cholesterol, mg/dL	Log10(x)	EXP	Nor	Id	-	0.53	0.19	0.38	0.64	-	-
Glucose, mg/L	$x^{3/1000}$	EXP	Nor	Id	-	0.95	0.48	0.21	0.86	0.16	-
FA, mEq/L	$1/x^{1/3}$	EXP	Nor	Id	-	0.16	0.06	0.49	0.19	-	-

[1]MPD = milkings per day; BW = body weight; TEM = total eating minutes; con., kg/45 kg milk = pellets, kg per 45 kg of milk; con., kg/45 kg ECM = pellets, kg per 45 of energy corrected milk; MY= milk yield; SNF = solids not fat; MUN = milk urea nitrogen; 4% FCM = 4% fat corrected milk; ALT = alanine aminotransferase; AST = aspartate transaminase; BHB = β-hydroxybutyrate; FA = fatty acids

[2]Transformation used for response variable x to produce empirically (Shapiro-Wilk test) or subjectively (histogram) Gaussian distributions

[3]Experimental (EXP; 4 to 29 days in milk, DIM) and carryover (CO; 30 to 90 DIM) phases

[4]Fixed effects included in respective models including the covariate (COV), treatment (TRT), the interaction of TRT and lactation group (LG; TRT × LG). the interaction of TRT and DIM (TRT × DIM), the interaction of LG and DIM (LG × DIM), and the interaction of TRT, LG, and DIM (3-way). The selected variance-covariance (VCOV) are also included for repeated measure models.

[5]Previous lactation 305 d mature equivalent milk production (PME305) was used as a covariate

[6]Variance covariance structures (VCOV) were selected that minimized the model Bayers information. VC = variance components; ar(1) = first order autoregressive; arh(1) = heterogeneous first order autoregressive
